# Supplementary material for: Pancreatic cancer is marked by complement-high blood monocytes and tumor-associated macrophages
Source: Life Sci Alliance. 2021 Mar 29;4(6):e202000935. doi: 10.26508/lsa.202000935 (PMC8091600; doi:10.26508/lsa.202000935)
Supplement: Supplementary file 3 [file LSA-2020-00935_TableS1.docx]

Supplementary Table 1. CyTOF antibodies

| **CyTOF**  **Antibody** | **Supplier** | **Clone** | **Dilution** | **Label** |
| --- | --- | --- | --- | --- |
| CD45 | Fluidigm | 30-F11 | 1:200 | 089Y |
| Ly-6G | Fluidigm | 1A8 | 1:400 | 141Pr |
| CD11b (Mac-1) | Fluidigm | M1/70 | 1:400 | 143Nd |
| CD4 | Fluidigm | RM4-5 | 1:200 | 145Nd |
| F4/80 | Fluidigm | BM8 | 1:100 | 146Nd |
| CD140a (PDGFRα) | Fluidigm | APA5 | 1:100 | 148Nd |
| CD19 | Fluidigm | 6D5 | 1:200 | 149Sm |
| Ly-6C | Fluidigm | HK1.4 | 1:500 | 150Nd |
| CD3e | Fluidigm | 145-2C11 | 1:100 | 152Sm |
| CD274 (PD-L1) | Fluidigm | 10F.9G2 | 1:100 | 153Eu |
| CD31 (PECAM-1) | Fluidigm | 390 | 1:100 | 165Ho |
| CD8a | Fluidigm | 53-6.7 | 1:200 | 168Er |
| CD206 (MMR) | Fluidigm | C068C2 | 1:200 | 169Tm |
| CD161 (NK1.1) | Fluidigm | PK136 | 1:100 | 170Er |
