# Supplementary material for: Pancreatic cancer is marked by complement-high blood monocytes and tumor-associated macrophages
Source: Life Sci Alliance. 2021 Mar 29;4(6):e202000935. doi: 10.26508/lsa.202000935 (PMC8091600; doi:10.26508/lsa.202000935)
Supplement: Supplementary file 6 [file LSA-2020-00935_TableS3.docx]

Supplementary Table 3. Antibodies used for immunostaining

| **Antibody** | **Supplier** | **Catalog Number** | **IHC**  **dilution** | **IF dilution** |
| --- | --- | --- | --- | --- |
| Alpha-smooth muscle actin | Sigma-Aldrich | A2547 | - | 1:1000 |
| CK19 (TromaⅢ) | Iowa Developmental Hybridoma Bank | - | - | 1:100 |
| F4/80 | Cell Signaling | 70076 | - | 1:200 |
| Ym1 | R&D Systems | AF2446 | 1:500 | - |
| C1q | abcam | ab71940 | - | 1:50 |
| E-cadherin | Cell Signaling | 14472 | - | 1:200 |
